# Molecular cancer classification using a meta-sample-based regularized robust coding method

**DOI:** 10.1186/1471-2105-15-S15-S2

**Published:** 2014-12-03

**Authors:** Shu-Lin Wang, Liuchao Sun, Jianwen Fang

**Affiliations:** 1 College of Computer Science and Electronics Engineering, Hunan University, Hunan, 410082, China; 2 Biometric Research Branch, Division of Cancer Treatment and Diagnosis, National Cancer Institute, Rockville, MD 20850, USA; 3 Applied Bioinformatics Laboratory, University of Kansas, Lawrence, KS 66045, USA

## Abstract

**Motivation:**

Previous studies have demonstrated that machine learning based molecular cancer classification using gene expression profiling (GEP) data is promising for the clinic diagnosis and treatment of cancer. Novel classification methods with high efficiency and prediction accuracy are still needed to deal with high dimensionality and small sample size of typical GEP data. Recently the sparse representation (SR) method has been successfully applied to the cancer classification. Nevertheless, its efficiency needs to be improved when analyzing large-scale GEP data.

**Results:**

In this paper we present the meta-sample-based regularized robust coding classification (MRRCC), a novel effective cancer classification technique that combines the idea of meta-sample-based cluster method with regularized robust coding (RRC) method. It assumes that the coding residual and the coding coefficient are respectively independent and identically distributed. Similar to meta-sample-based SR classification (MSRC), MRRCC extracts a set of meta-samples from the training samples, and then encodes a testing sample as the sparse linear combination of these meta-samples. The representation fidelity is measured by the *l*_2_-norm or *l*_1_-norm of the coding residual.

**Conclusions:**

Extensive experiments on publicly available GEP datasets demonstrate that the proposed method is more efficient while its prediction accuracy is equivalent to existing MSRC-based methods and better than other state-of-the-art dimension reduction based methods.

## Introduction

With the advance of DNA microarray and next-generation sequencing (NGS) technology [1], a large amount of gene expression profiles (GEP) data has been rapidly accumulated, which requires novel analysis method to deeply mine these big data to interpret such data to gain insight into the mechanism of tumor development. Since Golub *et al*. made use of gene expression profiling data, obtained using the DNA microarray technology, to classify acute myeloid leukemia (AML) and acute lymphocytic leukemia (ALL) [2], a great number of GEP-based cancer classification methods have been proposed for classifying cancer types or subtypes [3-6]. It has increasingly become clear that common machine learning methods such as support vector machine (SVM) [7,8] and artificial neural networks (ANN) [5,9] may not perform very well because of the curse of dimensionality, as the number of features (genes) is usually much higher than the number of samples in most GEP experiments. Therefore, the key task of GEP-based cancer classification should be the design of dimension reduction method to dramatically decrease the number of features in GEP data before constructing classification models.

Dimension reduction methods can be grouped into two categories: feature selection and feature reduction approaches. Feature selection methods [10], such as the heuristic breadth-first search algorithm, find as many optimal gene subsets as possible and further rank these genes to discover important cancer-related genes [11]. Feature extraction methods instead employ independent component analysis to model the gene expression data [12,13]. Gene selection methods do not alter the original representation of each gene, while feature extraction methods, which are based on projection, yield new variables that may reflect the intrinsic characteristics of original features. Other feature extraction methods such as principal component analysis (PCA)[14], linear discriminant analysis (LDA) [15], locally linear discriminant embedding (LLDE) [16], and partial least squares (PLS) [17] are also extensively applied to the dimensionality reduction of GEP. These methods can generally achieve satisfactory classification performance with the minimum dimension reduction. Both feature selection and feature extraction methods have their own advantages and disadvantages. For gene selection methods, their main advantage is that the selected genes may be related to the underlying mechanisms of cancer development. However, different gene selection methods may result in significantly different selected genes and therefore the interpretation of the results can be difficult. For the feature extraction methods, small dimension can be obtained by integrating original features. However, it is difficult to precisely interpret the biomedical meanings of derived features.

Machine learning based methods are also often called model-based methods because a predictive model is built for predicting the label of test sample. The model selection is a complex training procedure, which easily leads to over-fitting and decreased prediction performance. Recently, sparse representation (SR), a powerful data processing method that does not require model selection, has been extensively applied to face recognition [18,19] and further extended to cancer classification recently [20-22]. For example, Hang, *et al*. proposed a SR-based classification (SRC) method using $l_{1}$-norm minimization to classify cancer test sample. The approach models a classification problem as to find a sparse representations of test samples with respect to training samples [22]. They applied the proposed method to six cancer gene expression datasets and their experimental results demonstrated that the performance of the proposed method was comparable to or better than those of SVMs. Especially, the proposed method does not involve model selection and is robust to noise, outliers and even incomplete measurements. Zheng, *et al*. further presented a new SR-based method for GEP-based cancer classification, termed meta-sample-based SR classification (MSRC), where a set of meta-samples are extracted from training samples, and then a testing sample is represented as the linear combination of these meta-samples by $l_{1}$-regularized least square method [20]. Their experiments on publicly available GEP datasets have shown that MSRC is efficient for cancer classification and can achieve higher accuracy than many existing representative schemes such as SVM, SRC and least absolute shrinkage and selection operator (LASSO) algorithm. In addition, Gan *et al*. proposed a new classifier, meta-sample-based robust sparse representation classifier (MRSRC) based on the MSRC method, for cancer classification [21]. Their experiments show that these methods are efficient and robust.

Previous SR-based model assumes that the coding residual follows Gaussian or Laplacian distribution, which may not be effective for describing the coding residual in practical GEP datasets, and another problem is that the sparsity constraint on coding coefficients leads to the high computational cost of SRC method. To deal with the problem, Yang *et al*. proposed a new coding model, namely regularized robust coding (RRC) for face recognition [23]. Here, we present a meta-sample-based regularized robust coding classification (MRRCC) method, a novel and effective cancer classification technique combining the ideas of meta-sample-based and RRC methods. A meta-sample can be represented as a linear combination of a set of training samples, which may capture the intrinsic structures of these data. The coefficient vector of a meta-sample may have only a few nonzero elements. The expression patterns over the meta-samples can reflect the gene expression patterns. Test samples belonging to the same subclass will have similar sparse representation, while different subclass would result in different sparse representations [22]. Our extensive experiments on cancer datasets show that MRRCC can achieve higher classification accuracy but with lower time complexity, compared with other SR-based methods and dimension reduction-based methods.

## Methods

### Description of SR-based problem

Let $G=\left\{g_{1},\cdots \;,g_{n}\right\}$ be a set of genes and $S=\left\{s_{1},\cdots \;,s_{m}\right\}$ be a set of samples. $\left|G\right|=n$ denotes the number of genes, and |S|=m denotes the number of samples. The corresponding GEP data can be represented as a matrix $X={\left(x_{i,j}\right)}_{nm},1\leq i\leq n,1\leq j\leq m$, where $x_{i,j}$ is the expression level of gene $g_{i}$ in sample $s_{j}$. Usually  n is much bigger than  m for a typical GEP dataset. Each vector $s_{i}$ in the gene expression matrix can be regarded as a point in *n*-dimensional space. Each of the *m *columns consists of an *n*-element expression vector for a single sample. Let $L=\left\{c_{1},\cdots \;,c_{k}\right\}$ denote the label set and $\left|L\right|=k$ denote the number of subclasses. Because the subclass of each sample is known, $S\times L=\left\{\left(s_{i},l_{i}\right)|s_{i}\in R^{n},l_{i}\in R^{n},l_{i}\in L,i=1,2,\cdots \;,m\right\}$ denotes the labeled sample space. The whole sample set  X is randomly split into two disjoint parts: training set  A and test set  B. Generally, the SR-based problem could be represented as

(1) $$\underset{\alpha }{\text{min}}||\alpha |{|}_{1},\;\text{s}.\text{t}.||y-A\alpha |{|}_{2}^{2}\leq \varepsilon$$

where  y is a given test sample,  A represents all training samples,  α is the coding vector of  y with respect to  A, and  ε is a small positive constant. By coding the test sample  y as a sparse linear combination of the training samples via Eq. (1), SR-based classifier assigns the label to the test sample  y based on the predictions which subclass can produce the least reconstruction error.

### Analysis flowchart of cancer GEP data

**Figure 1:**
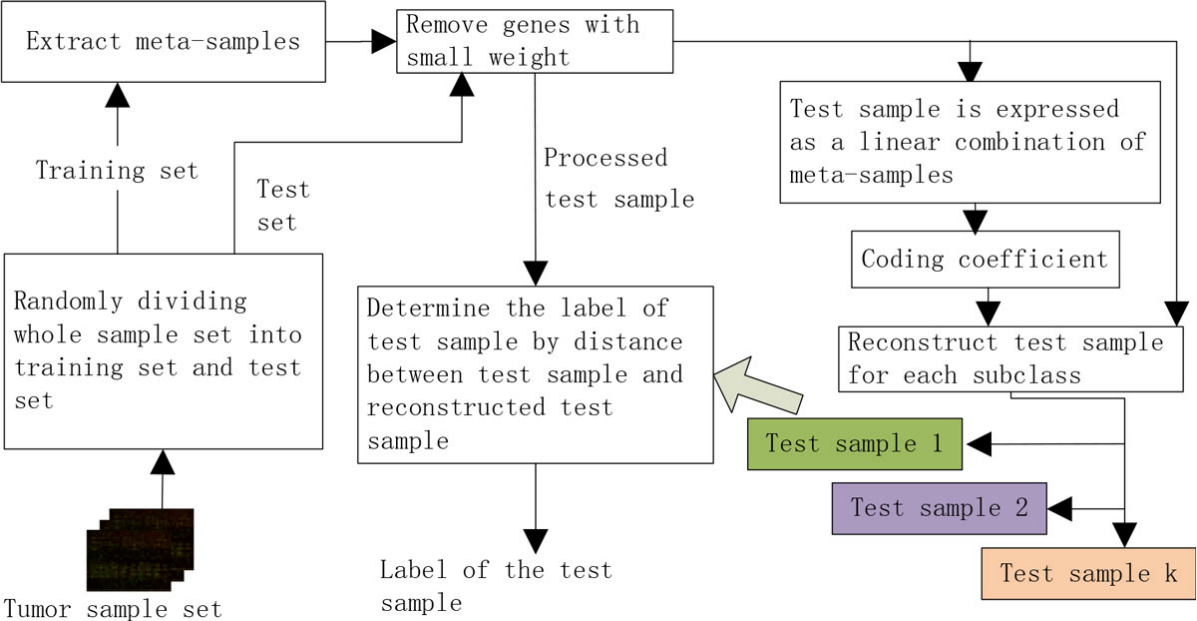
**The analysis flowchart of cancer GEP data using SR-based methods for predicting cancer types**.

The analysis flowchart of the meta-sample-based SR method is different from those of traditional model-based and template-based methods (Figure 1). The classification models of model-based methods use the training set to predict the labels of test samples, while template-based methods create a template for each subclass using training set and then compare a test sample to the templates in order to determine the label of the test sample [3]. Although there is similarity between the analysis flowcharts of meta-sample-based SR method and template-based one, there is a major difference (Figure 1). The reconstructed test samples of the meta-sample-based SR method are relevant to not only the training set but also the original test sample, while the templates of template-based methods are only relevant to the training set. The flowchart of analysis of the meta-sample-based SR method includes five steps:

1) The whole sample set is randomly split into two disjoint parts: training set and test set, and then the meta-samples are extracted only from the training set using singular value composition (SVD).

2) The weight of each gene is calculated according to a weight function, and the genes with lower weight are removed in a test sample $T_{o}$ and all meta-samples.

3) The test sample $T_{o}$ is represented as a linear combination of all meta-samples, and the coding coefficient of the test sample $T_{o}$ can be obtained by using RRC.

4) We can reconstruct the test sample for each subclass by using the meta-samples and the coding coefficient of the original test sample $T_{o}$, and the reconstructed test samples (the test sample 1, test sample 2,..., test sample  k) are denoted by $T_{1},T_{2},\ldots ,T_{k}$, where  k denotes the number of subclasses in original dataset.

5) The distance between the processed test sample and each reconstructed test sample $T_{i},1\leq i\leq k$ is calculated, and the original test sample $T_{o}$ is assigned to the subclass with minimal distance.

### Construct meta-samples

The meta-sample extracted from GEP data is commonly defined as a linear combination of all training samples. In this paper, a set of meta-sample is extracted from all training samples of one cancer type. We find that meta-sample can capture the structures to the data and offer biological insight. on the other hand, the linear combination of the meta-samples can roughly estimate genetic expression pattern of gene data [24]. Alter, *et al*. used singular value decomposition (SVD) to transform GEP data from a space of genes  × arrays to the diagonal ''eigengenes  × eigenarrays'' space [25], where the eigenarrays (or eigengenes) are the unique orthonormal superpositions of the arrays (or genes). In our approach, we can decompose the gene expression data set matrix  A into two matrices:

$$A_{i}=M_{i}\times V_{i},\;1\leq i\leq k$$

where matrix $A_{i}$ is of size $n\times m_{i}$, the matrix $M_{i}$ is of size $n\times q_{i}$ and the matrix $V_{i}$ is of size $q_{i}\times m_{i}$. Each of $q_{i}$ columns in matrix $M_{i}$ is defined as a meta-sample of the  i-th subclass. Each of $m_{i}$ columns in matrix $V_{i}$ represents the meta-sample expression pattern of the corresponding samples. $D=\left[M_{1},\ldots M_{i},..M_{k}\right]$ denotes the constructed meta-sample set.

### Calculating coding coefficient using RRC

The meta-sample set $D=\left[M_{1},\ldots M_{i},..M_{k}\right]$ can be rewritten as $D=\left[r_{1};\ldots r_{i};..r_{n}\right]$, where $r_{i}$ denotes the expression level of the  i-th gene in all meta-samples. Let $y=\left[y_{1};\ldots y_{i};\ldots y_{n}\right]$ denote a test sample, where $y_{i}$ is the expression level of the  i-th gene. We can consider the cancer classification from the view point of Bayesian estimation, especially the maximum a posterior (MAP) estimation. By using the Bayesian formulation, we can calculate the coding coefficient by the following formula [23]

(2) $$\hat{\alpha }=\text{arg}{\text{min}}_{\alpha }\left\{\sum_{i=1}^{n}\rho_{\theta }\left(y_{i}-r_{i}\alpha \right)+\sum_{j=1}^{m}\rho_{o}\left(\alpha_{j}\right)\right\}$$

where $\rho_{\theta }\left(e\right)=-lnf_{\theta }\left(e\right)$ and $\rho_{o}\left(\alpha \right)=-lnf_{o}\left(\alpha \right)$. The coding residual $e=y-D\alpha =\left[e_{1};e_{2};\ldots ;e_{n}\right]$ are with the probability density function (PDF) $f_{\theta }\left(e_{i}\right)$ and the coding vector $\alpha =\left[\alpha_{1};\alpha_{2};\ldots ;\alpha_{m}\right]$ are with PDF$f_{o}\left(\alpha_{j}\right)$. Generally, we assume that the unknown PDF $f_{\theta }\left(e\right)$ are symmetric, differentiable and monotonic. Therefore, $\rho_{\theta }\left(e\right)$ has following properties: (1) $\rho_{\theta }\left(0\right)$is the global minimal of $\rho_{\theta }\left(z\right)$; (2)$\rho_{\theta }\left(z\right)=\rho_{\theta }\left(-z\right)$; (3) if $\left|z_{1}\right|<|z_{2}|$, we can get $\rho_{\theta }\left(z_{1}\right)<\rho_{\theta }\left(z_{2}\right)$. Without loss of generality, we can let $\rho_{\theta }\left(0\right)=0$.

There are two key issues in solving the RRC model. The first one is how to determine the distribution $\rho_{\theta }$. The second one is how to minimize the energy function. The RRC model in Eq. (2) can be approximated as follows.

(3) $$\hat{\alpha }=\text{arg}{\text{min}}_{\alpha }\left\{\frac{1}{2}||W^{1\;/\;2}\left(y-D\alpha \right)|{|}_{2}^{2}+\sum_{j=1}^{m}\rho_{o}\left(\alpha_{j}\right)\right\}$$

where  W is a diagonal matrix.

(4) $$W_{i,i}=\omega_{\theta }\left(e_{0,i}\right)={\rho^{\prime }}_{\theta }\left(e_{0,i}\right)/e_{0,i}$$

where $W_{i,i}$ is the weight value of each gene. Thus the minimization problem of the RRC model can be transformed into calculating the diagonal weight matrix  W.

The logistic function has the same properties as the hinge loss function in SVM [26], so we choose it as the weight function.

(5) $$\omega_{\theta }\left(e_{i}\right)=\text{exp}\left(\mu \delta -\mu e_{i}^{2}\right)/\left(1+exp\left(\mu \delta -\mu e_{i}^{2}\right)\right)$$

where  μ and  δ are two positive constants. Parameter  μ controls the decreasing rate from 1 to 0, and  δ controls the location of demarcation point. To make $\omega_{\theta }\left(0\right)$ close to 1, let the value of μδ be big enough. According to Eq. (4), Eq. (5) and$\rho_{\theta }\left(0\right)=0$, we can get

(6) $$\rho_{\theta }\left(e_{i}\right)=\frac{-1}{2\mu }\left(\text{ln}\left(1+\text{exp}\left(\mu \delta -\mu e_{i}^{2}\right)\right)-ln\left(1+\text{exp}\mu \delta \right)\right)$$

For cancer classification, the coding coefficients associated with the dictionary atoms from the same subclass would have big absolute values. However, we do not know which subclass the testing sample will belong to. Therefore, we actually assume that the coding coefficient $\alpha_{j}$ follows generalized Gaussian distribution (GGD). So we can obtain the following formula.

(7) $$f_{o}\left(\alpha_{j}\right)=\beta \text{exp}\left\{-{\left(|\alpha_{j}|/\sigma_{\alpha }\right)}^{\beta }\right\}/\left(2\sigma_{\alpha }\Gamma \left(1/\beta \right)\right)$$

where  Γ is the gamma function.

The RRC model has two vital cases when  β is set as two specific values [23].

When β=1, GGD degenerates to Laplacian distribution, and the RRC model will become

(8) $$\hat{\alpha }=\text{arg}{\text{min}}_{\alpha }\left\{W^{1\;/\;2}{\left(y-D\alpha \right)}_{2}^{2}+\lambda \alpha_{1}\right\}$$

When β=2, GGD degenerates to Gaussian distributon, and the RRC model will become

(9) $$\hat{\alpha }=\text{arg}{\text{min}}_{\alpha }\left\{W^{1\;/\;2}{\left(y-D\alpha \right)}_{2}^{2}+\lambda \alpha_{2}^{2}\right\}$$

### Iteratively reweighted regularized robust coding algorithm

Iteratively reweighted regularized robust coding (IR^3^C) algorithm was designed by Yang, *et al*. to solve the RRC model efficiently [23]. The overall procedure of the algorithm is as follows.

**Input**: Normalized test sample  y with unit $l_{2}$-norm; meta-sample set  Dextracted from original training samples; $\alpha^{\left(1\right)}$.

**Output**:  α

t=1; // *t *denotes the iterative times.

1. Compute the gene residual $e^{\left(t\right)}=y-D\alpha^{\left(t\right)}$

where $\alpha^{\left(1\right)}=\left[\frac{1}{m};\frac{1}{m};\ldots ;\frac{1}{m}\right],$ and $\text{D}\alpha^{\left(1\right)}$ is the mean of all meta-samples.

2. Estimate weight value of each gene as

$$\omega_{\theta }\left(e_{i}^{\left(t\right)}\right)=1/1+\text{exp}\left(\mu {\left(e_{i}^{\left(t\right)}\right)}^{2}-\mu \delta \right)$$

where  μ and  δ would be estimated in each iteration and  δ is associated with residual.

3. Weighted regularized robust coding coefficient:

$\alpha^{\text{*}}=\text{arg}\underset{\alpha }{\text{min}}\left\{\frac{1}{2}||{\left(W^{\left(t\right)}\right)}^{0.5}\left(y-D\alpha \right)|{|}_{2}^{2}+\sum_{j=1}^{m}\rho_{o}\left(\alpha_{j}\right)\right\}$; //Assume the $\alpha_{j}$ follows generalized Gaussian distribution.

4. Update the robust coding coefficients.

If t=1, $\alpha^{\left(t\right)}=\alpha^{*}$;

If t>1, $\alpha^{\left(t\right)}=\alpha^{\left(t-1\right)}+v^{\left(t\right)}\left(\alpha^{*}-\alpha^{\left(t-1\right)}\right)$; //where $0<v^{\left(t\right)}\leq 1$ is a suitable step size. $v^{\left(t\right)}$could be searched from 1 to 0 by the standard line-search process [27].

5. Reconstruct the test sample by coding coefficient and all meta-samples

$y_{rec}^{\left(t\right)}=D\alpha^{\left(t\right)}$, and let t=t+1.

6. Return to the step 1 until the condition of convergence $||W^{\left(t\right)}-W^{\left(t-1\right)}|{|}_{2}/||W^{\left(t-1\right)}|{|}_{2}<\varphi$ ( φ is a small positive scalar) is met, or reached the maximal number of iteration.

Algorithm end.

When the algorithm converges, we can use the same classification method as SRC to classify test sample.

(10) $$identity\left(y\right)=\text{arg}{\text{min}}_{d}\left\{l_{d}\right\}$$

where $l_{d}=||W_{final}^{\frac{1}{2}}\left(y-D_{d}{\hat{\alpha }}_{d}\right)|{|}_{2}$, $D_{d}$ is the meta-sample set associated with *d*-th subclass, ${\hat{\alpha }}_{d}$ is the final coding vector associated with *d*-th subclass, and $W_{final}$ is the final weight matrix.

When β=1, the time complexity of IR^3^C is $O\left(tm^{2}n\right)$, where  n is the number of genes,  m is the number of meta-samples, and  t is the iteration times. When β=2, the time complexity of IR^3^C is $O\left(tk_{1}mn\right)$, where $k_{1}$ is the iteration number in conjugate gradient solution. The time complexity of IR^3^C with β=1 or β=2 is much lower complexity than SRC whose time complexity is $O\left(m^{2}n^{1.5}\right)$[23].

In literature [23] the RRC model with β=1 is called as RRC_L1 and the RRC model with β=2 is called as RRC_L2. However, in our method the input  D of IR^3^C is actually a set of meta-samples which are extracted by SVD from the original training set, so we call our methods as MRRCC1 (the meta-sample-based regularized robust coding classification 1) and MRRCC2 (the meta-sample-based regularized robust coding classification 2) corresponding to the two cases RRC_L1 and RRC_L2, respectively.

## Experiments

### Cancer datasets

GEP data can be obtained by two technologies, DNA microarray and next-generation sequencing (NGS) technologies. In our experiments five microarray and four NGS cancer datasets are used to evaluate the proposed method (Table 1). The five microarray datasets include Diffuse Large B-cell Lymphomas (DLBCL) [28], Acute Lymphoblastic Leukemia (ALL) [29], GCM [30], Lung cancer (Lung) [31], and MLL [32]. The DLBCL dataset contains two subclasses, i.e., DLBCL and Follicular Lymphoma (FL). The ALL dataset totally contains 248 samples that belong to six cancer subtypes: BCR-ABL, E2A-PBX1, Hyperdip>50, MLL, T-ALL and TEL-AML1. The GCM dataset consists of fourteen different cancer types. The Lung cancer dataset contains four lung cancer types and one normal tissue type (i.e., five subclasses in total). The MLL dataset contains 72 samples from three subtypes or subclasses, i.e., MLL, AML and ALL.

**Table 1:** The summary of the eight cancer datasets.

Types	Datasets	#Samples	#Genes	#Subclasses(*K*)
Microarray	DLBCL	77	7,129	2
	ALL	248	12,626	6
	GCM	190	16,063	14
	Lung	203	12,601	5
	MLL	72	7,129	3

NGS	BRCACancer	216	20531	2
	KIRCCancer	130	20531	2
	LUADCancer	110	20531	2
	THCACancer	112	20531	2

The four NGS datasets are downloaded from the web site: The Cancer Genome Atlas (TCGA) (http://cancergenome.nih.gov/). They include Breast invasive carcinoma (called as BRCACancer), Kidney renal clear cell carcinoma (KIRCCancer), Lung adenocarcinoma (LUADCancer), and Thyroid carcinoma (THCACancer). All samples are matched cancer and normal tissue samples.

## Parameter selection

There are two parameters, namely  μ and δ, in weight function Eq. (5). We calculate the  δ value as follows. Let *l=*⌊τm⌋, where τ∈(0,1). Parameter  δ can be obtained by the following formula.

(11) $$\delta =\gamma_{1}{\left(e\right)}_{l}$$

where the vector $e\in R^{n}$, $\gamma_{1}{\left(e\right)}_{q}$ is the  q-th largest element of the set $\left\{e_{j}^{2},\text{j}=1,\ldots ,\text{n}\right\}$. Parameter  μ is used to control the decreasing rate of the weight $W_{i,i}$. We can simply set μ=s/δ, where s=8 is defined as a constant. So the  δ value, estimated by according to Eq. (11), is a very important parameter to distinguish outlier genes. The selection of parameter  τ will be further determined by our experiments.

**Figure 2:**
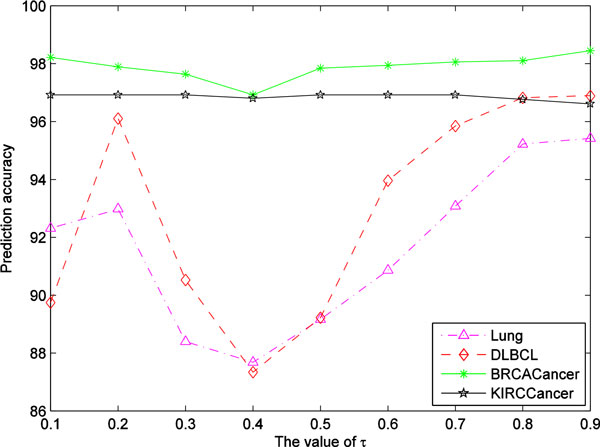
The prediction accuracy on the four data sets varying with different  τ value.

Figure 2 shows the 10-fold cross validation prediction accuracy varying with  τ value increasing from 0.1 to 0.9 by 0.1 on four cancer datasets (two microarray datasets and two NGS datasets), from which we can see that the optimal prediction accuracy can be achieved on the four datasets when  τis set to 0.9. So it is appropriate that  τis fixed to 0.9 in our experiments. Furthermore, we find that the prediction accuracy on two microarray datasets is greatly affected by  τ values, while the prediction accuracy on two NGS datasets is less affected by  τ values, suggesting that the two microarray datasets are noisier than the two NGS datasets.

## Comparison with other SR-based methods

**Figure 3:**
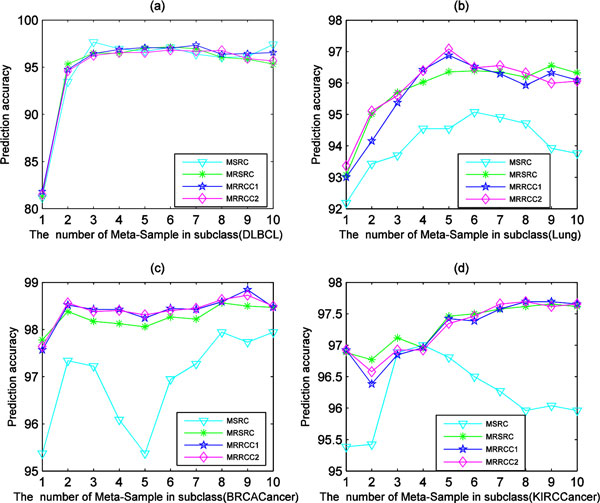
**The prediction accuracy of the four methods varying with different number of meta-samples on the four datasets**.

The prediction accuracy of the meta-sample-based methods is greatly affected by the number of meta-samples extracted using SVD. Figure 3 shows the prediction accuracy of the four SR-based methods (MSRC, MRSRC, MRRCC1 and MRCC2) varying with the number of meta-samples on four datasets, respectively. And Figure 3 shows that no fixed number of meta-samples can consistently achieve the optimal performance of meta-sample-based methods. Therefore, the meta-sample-based methods require the process of optimizing the number of meta-samples. Here two-layer 10-fold cross-validation is used to evaluate the performance of the SR-based methods. The inner layer 10-fold cross-validation is used to determine the optimal number of meta-samples for training in outer layer 10-fold cross-validation, and the outer layer 10-fold cross-validation is used to evaluate the classification performance of SR-based methods. The classification accuracy obtained by five SR-based methods on the nine cancer datasets are shown in Table 2. It is clear that our methods MRRCC1 and MRRCC2 are equivalent to other three SR-based methods in optimal prediction accuracy on eight datasets except on GCM dataset.

**Table 2:** The classification accuracy obtained by five SR-based methods on the nine cancer datasets.

Types	Datasets	SRC	MSRC	MRSRC	MRRCC1	MRRCC2
Microarray	DLBCL	94.75	96.10	94.81	**97.40**	94.81
	All	97.70	97.18	97.81	96.77	**97.18**
	GCM	82.93	82.32	78.79	79.80	78.79
	Lung	94.53	95.57	96.55	**96.55**	**96.55**
	MLL	96.31	95.83	98.61	97.22	**98.61**

NGS	BRCACancer	96.76	95.83	99.07	**99.07**	**99.07**
	KIRCCancer	95.92	95.38	96.92	**96.92**	**96.92**
	LUADCancer	94.91	99.09	99.09	**100**	**99.09**
	THCACancer	93.30	87.50	95.54	92.86	**95.54**

## Comparison with dimension reduction-based methods

A two-stage method can be used to reduce the dimensionality of dataset before classification. The first stage is a process of adopting a gene filter method such as KWRST (Kruskal-Wallis rank sum test) [33] or Relief-F [34] to initially select a small set of differentially expressed genes. The second stage is a process of adopting a feature extraction method to further reduce the dimensionality of the dataset. Our previous studies have shown that the predication accuracy of two-stage method is influenced by many factors such as normalization method, gene filter method, feature extraction method, classification method, the number of genes selected and the number of features extracted as well as different division of training set and test set, etc. [35]. In our experiments, training sets and test sets are normalized by samples using the z-score normalization method. KWRST is used to filter genes and 300 top-ranked genes are initially selected. The five feature extraction methods (PCA, LDA, ICA, LLDE, and PLS) are used to reduce the dimensionality of dataset. *K*-nearest neighbor (KNN), one of simplest classification methods, with correlation distance is used to classify cancer samples (here 5 nearest neighbors are used). For LDA method and the datasets with two subclasses, Euclidean distance is used because only one feature is extracted. To avoid over-fitting, before classification we extract only 5 features using these feature extraction methods except LDA whose number extracted is K-1. We call these methods as PCAKNN, LDAKNN, ICAKNN, LLDEKNN, and PLSKNN, respectively.

**Figure 4:**
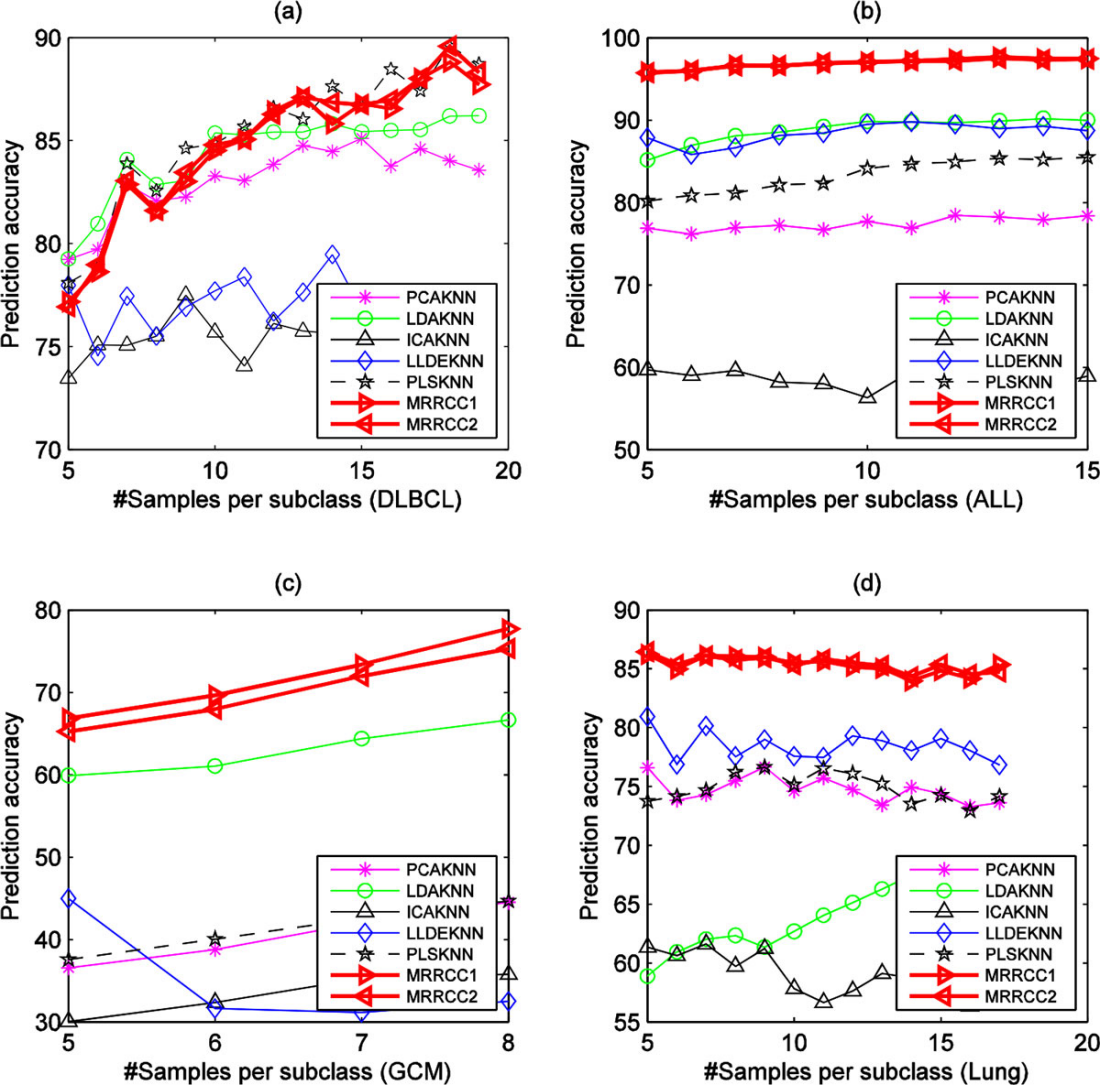
**The performance of seven methods varying with the number of genes on the four microarray GEP datasets**.

**Figure 5:**
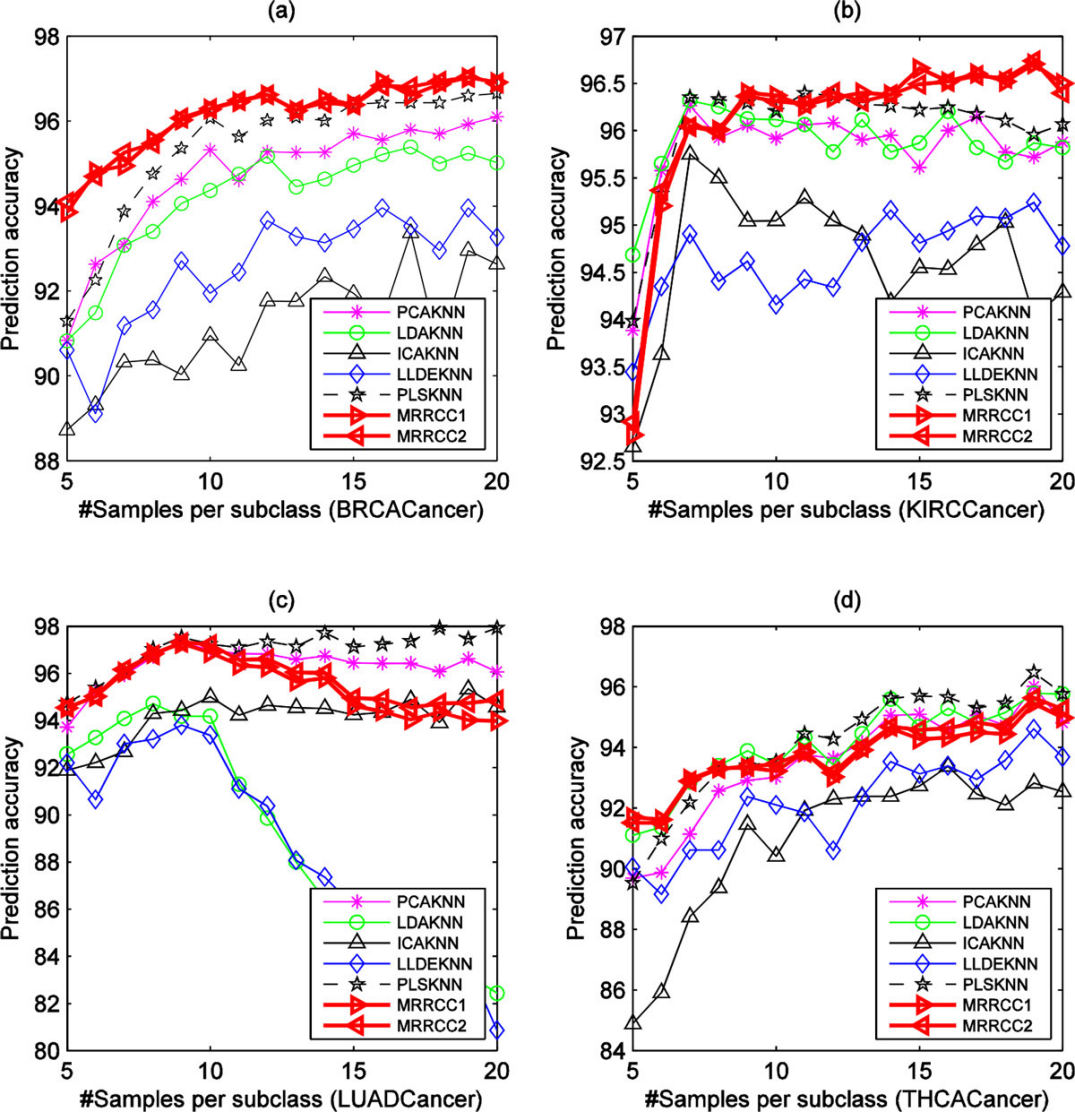
**The performance of seven methods varying with the number of genes on the four NGS GEP datasets**.

Experiments indicate that the different divisions of training sets and test sets can also greatly affect the classification performance. In our experiments, the Balance Division Method (BDM) is used to divide each original dataset into balanced training sets and test sets [4]. For the BDM,  q samples from each subclass of the original dataset are randomly selected and used as a training set, while the remaining samples are used as test set. Here the limits of  q value ranges from 5 to $\left|c_{\text{min}}\right|$, where $c_{\text{min}}$ denotes the subclass set with minimum number of samples in the original dataset, i.e., $\underset{c_{i}}{c_{\text{min}}\text{= argmin}}\left(\left|c_{i}\right|\right),\;1\leq i\leq K$, where  k denotes the number of subclass in dataset. We set  q value to 20 when $|c_{\text{min}}|>20$,. For each  q value, the statistical mean of prediction accuracies obtained on 100 randomizations of training set and test set are calculated for each method. Figure 4 and Figure 5 show the performance of eight methods varying with different numbers of training samples per subclasses on four microarray datasets and four NGS datasets, respectively. The experimental results indicate that the performance of MRRCC1 and MRRCC2 are almost the same for all but the GCM dataset. Generally, our methods are superior to other five methods in predication accuracy not only on the four microarray datasets but also on the four NGS datasets. On the LUADCancer and THCACancer datasets the performance of our methods is slightly worse than PLSKNN in prediction accuracy when the number of the samples per subclass in training sets is greater than 10.

## Conclusions

With the development of microarray and NGS technologies, a huge amount of GEP data is rapidly accumulated, demanding more efficient analysis methods to analyze these data. In this paper we present a novel meta-sample-based regularized robust coding for cancer classification (MRRCC) that firstly represents each test sample as a linear combination of all meta-samples which are extracted from the original training set using SVD. The coefficient vector is then obtained by $l_{2}$-regularized least square that is as powerful as *l*1-norm regularization but the former has much lower computational cost [23]. The experimental results have demonstrated that MRRCC can achieve higher classification accuracy with lower computational cost than previous state-of-the-art solutions such as SRC, MSRC and MRSRC, as well as many dimension reduction based classification methods.

## Competing interests

The authors declare that they have no competing interests.

## Authors' contributions

Shu-Lin Wang designed the framework of analysis, performed the partial experiments, and drafted the manuscript. Liuchao Sun also performed the partial experiments, and Jianwen Fang analyzed the numerical results and revised the manuscript. All authors read and approved the final manuscript.
